# Structure of variability in scanning movement predicts braille reading performance in children

**DOI:** 10.1038/s41598-021-86674-5

**Published:** 2021-03-30

**Authors:** Tetsushi Nonaka, Kiyohide Ito, Thomas A. Stoffregen

**Affiliations:** 1 Graduate School of Human Development and Environment, Kobe University, Tsurukabuto 3-11, Nada-ku, Kobe, 6578501 Japan; 2 School of Systems Information Science, Future University Hakodate, Hakodate, Japan; 3 School of Kinesiology, University of Minnesota, Minneapolis, MN USA

**Keywords:** Psychology, Human behaviour, Learning and memory, Sensorimotor processing, Sensory processing, Motor control, Touch receptors

## Abstract

Among children learning to read braille, we asked whether the quantitative kinematics of scanning movements of the reading finger would be related to the proficiency of braille reading. Over a period of 12 months, we recorded the position and orientation of the reading fingers of eight congenitally or early blind children. We found that the strength of long-range power-law temporal correlations in the velocity fluctuations increased with performance in braille reading. In addition, we found that the variability of the angular orientation of the reading finger that affects the contact region on the fingerpad was negatively related to braille reading performance. These results confirm that the quantitative kinematics of finger scanning movements were related to functional performance in braille reading. The results add to the growing body of evidence that long-range temporal correlations in exploratory behavior can predict perceptual performance, and that scanning movements that center important tactile information on the small, high resolution area contribute to the pickup of information.

## Introduction

Braille is a form of reading that depends upon active touch: To read braille, the reader actively moves one or more fingers across the printed array of raised dots. During braille reading, variations in stimulation of the skin arise from the interaction between the printed dots and the reader’s movements^1–7^. For this reason, braille reading can be understood as an instance of active touch, or haptics^8–17^. Active movement also is used in visual reading of ordinary text, as gaze shifts across the page. However, visual reading differs from braille reading in an important way. Visual reading comprises alternation in gaze between shifts (including ocular saccades) and fixations. The apprehension of words occurs only during fixations^18^. By contrast, in braille reading there are no fixations: Apprehension of letters and words occurs only during scanning, that is, while the fingers are in motion^1–3^. Most braille is printed in horizontal rows, and so braille reading typically consists of lateral translation of one or more fingers along each line.

### Kinematics of the reading finger

One aspect of braille reading that has gained increasing attention concerns the quantitative details of finger movements in scanning. This area of research has been transformed as technology has made it possible to examine behavior in greater and greater detail. Early studies were limited to visual coding of video recordings^19–21^. The advent of technologies that permit direct measurement of finger kinematics is transforming our understanding of braille reading. Previous studies of finger kinematics in braille reading have focused on linear measures derived from spatial position, such as mean velocity and mean squared deviation from the average computed over a single, chosen time scale^22,23^. In those studies, fluctuations in finger movement were interpreted as noise in the neuromuscular system. However, it can be argued that such fluctuations play a functional role in enhancing the sensitivity and adaptability of sensorimotor systems^24–32^.

The kinematic characteristics of scanning movements of the reading finger may be related to reading performance. Casual observation suggests that skilled braille reading is characterized by “smooth, light, even scanning movements of the hands over the text (p.445)^2^”. Yet previous research demonstrates that movements that are visibly smooth typically contain subtle variations in lateral velocity of the finger on the page^22,23^. The possibility that movement fluctuations might contribute to the efficiency of braille reading has not been tested.

In the present study, we examined the quantitative kinematics of the reading finger among children who were learning to read braille. We related these kinematics to individual variations in reading performance. We asked whether and how the kinematics of finger movement would change over time as children learned to read braille.

We focused on two aspects of the structure of variability that have not been evaluated in previous research: (1) the temporal correlation structures of the velocity variation of the reading finger, and (2) variability in the orientation of the reading finger, which reflects the orientation of the fingerpad to the braille dots. We evaluated the temporal structure of a first-order differentiation of lateral scanning trajectories using the Hurst exponent estimated by detrended fluctuation analysis (DFA)^33^. DFA has been used in assessments of many aspects of animate movement. Common examples include research on dynamic touch^27–29^ and gaze behavior^34,35^. The Hurst exponent (*H*) represents the power-law relation between the magnitude of fluctuations in lateral finger displacement and the time scale over which those fluctuations are measured^36^. For uncorrelated random noise or anti-correlated series, *H* ≤ 0.5, but when fluctuations are temporally long-range correlated with systematically larger fluctuations at longer time scales and smaller fluctuations at shorter time scales, the value of *H* approaches 1. Studies of exploratory movement in manual wielding have shown that the strength of long-range temporal correlations, as measured by the Hurst exponent, predicted the accuracy of perceptual performance and the efficiency of perceptual learning^27–29^. Based on these previous findings, we hypothesized that the strength of long-range temporal correlations in scanning movements of the reading finger would correlate with braille reading performance.

The angular orientation of the reading finger may be controlled so as to center important tactile information on the most sensitive areas of the fingerpad. Such centering could resemble other cases in which perceivers orient the most sensitive portions of perceptual systems toward items of interest^37–41^. Braille readers might likewise hit upon a similar solution of orienting the small, high resolution area of the reading finger for the discrimination of tactile information. Accordingly, we hypothesized that the angular orientation of the reading finger that keeps a certain region of the reading finger in contact with braille dots would predict performance in braille reading.

**Figure 1:**
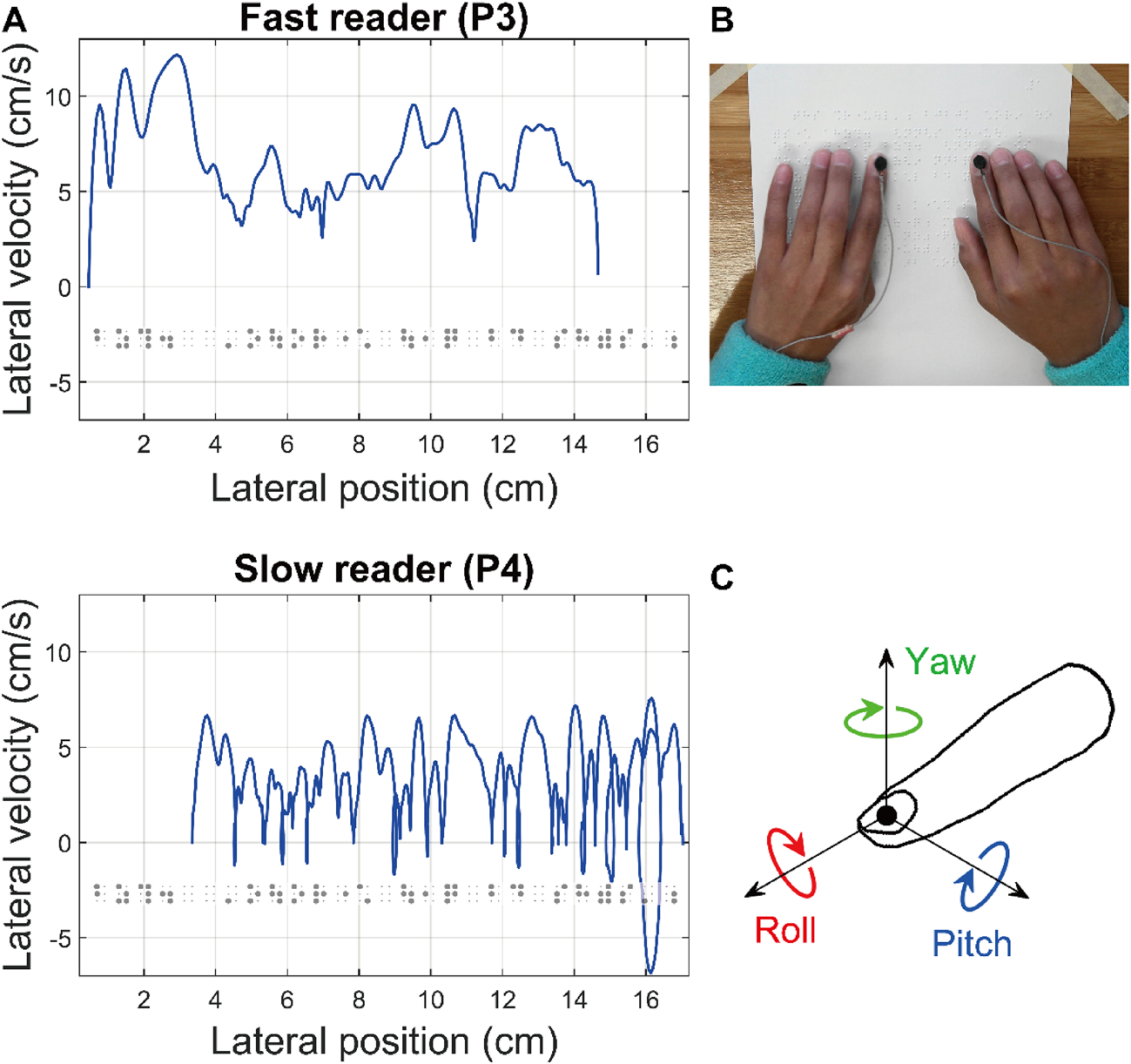
(**A**) Examples of individual lateral velocity traces as a function of the dominant reading finger’s lateral position on a single line of text (shown as rendered in contracted braille) of a fast reader (top, P3) and a slow reader (bottom, P4: In this trial, P4 skipped the first word of the line). (**B**) Experimental set-up. An electromagnetic micro-sensor was attached on the nail of each index finger of the participant reading braille. (**C**) Representation of the three angular displacements in the sensor attached on the nail of the index finger.

In a longitudinal field study, we recorded the movement of reading fingers of blind school children (Fig. 1). We followed the students over the course of one year, with repeated testing at 3-month intervals. We sought to document the characteristics of scanning movements of the dominant reading finger of braille readers (see Fig. 1A for examples), and the relation of these movements to braille reading performance. We did this by characterizing the difference in the movement of the reading finger between slow and fast readers, between trials within each session, and across testing sessions (Table 1, see “Methods” section). In addition, we predicted that reading performance would be related to the kinematics of the reading finger. Using a growth curve modelling technique^28,42^, we aimed to test two specific hypotheses. First, we predicted that reading performance would be related to strength of long-range temporal correlations in lateral scanning movements of the dominant reading finger. Second, we predicted that reading performance would be related to variability in the orientation of the dominant reading finger.

**Table 1:** Definition of the variables used for the analysis of braille reading behavior.

	Definition
**Independent variables**	
Repetition	Repetition number (1st or 2nd reading)
Month	Recording session month (April, July, October, January)
**Dependent variables (computed for each line of braille texts)**	
Reading speed	Number of braille characters orally read per each second (cps)
Finger velocity	Mean instantaneous finger velocity (cm/s)
No. zero-crossings	Number of acceleration zero-crossings per each cm travelled
No. reversals	Number of transitions from positive to negative velocity in the left–right dimension over more than 0.5 cm which is followed by a negative-to-positive transition (per line)
SD roll	Angular standard deviation of roll of the finger orientation (radians)
SD pitch	Angular standard deviation of pitch of the finger orientation (radians)
SD yaw	Angular standard deviation of yaw of the finger orientation (radians)
Hurst exponent (H)	Scaling exponent indicating temporal correlations in finger displacement series

## Results

The total number of lines across all the eight participants that contributed the analysis was 574 lines (13,681 characters). P4 was unable to read braille sentences at the first recording, and the data of the first session does not include P4. P8 read only a few lines in the first and second session, which she read only once without repetition, and the analysis of the first and second sessions only includes the data of a few lines from the first reading by P8.

### Reading performance

**Figure 2:**
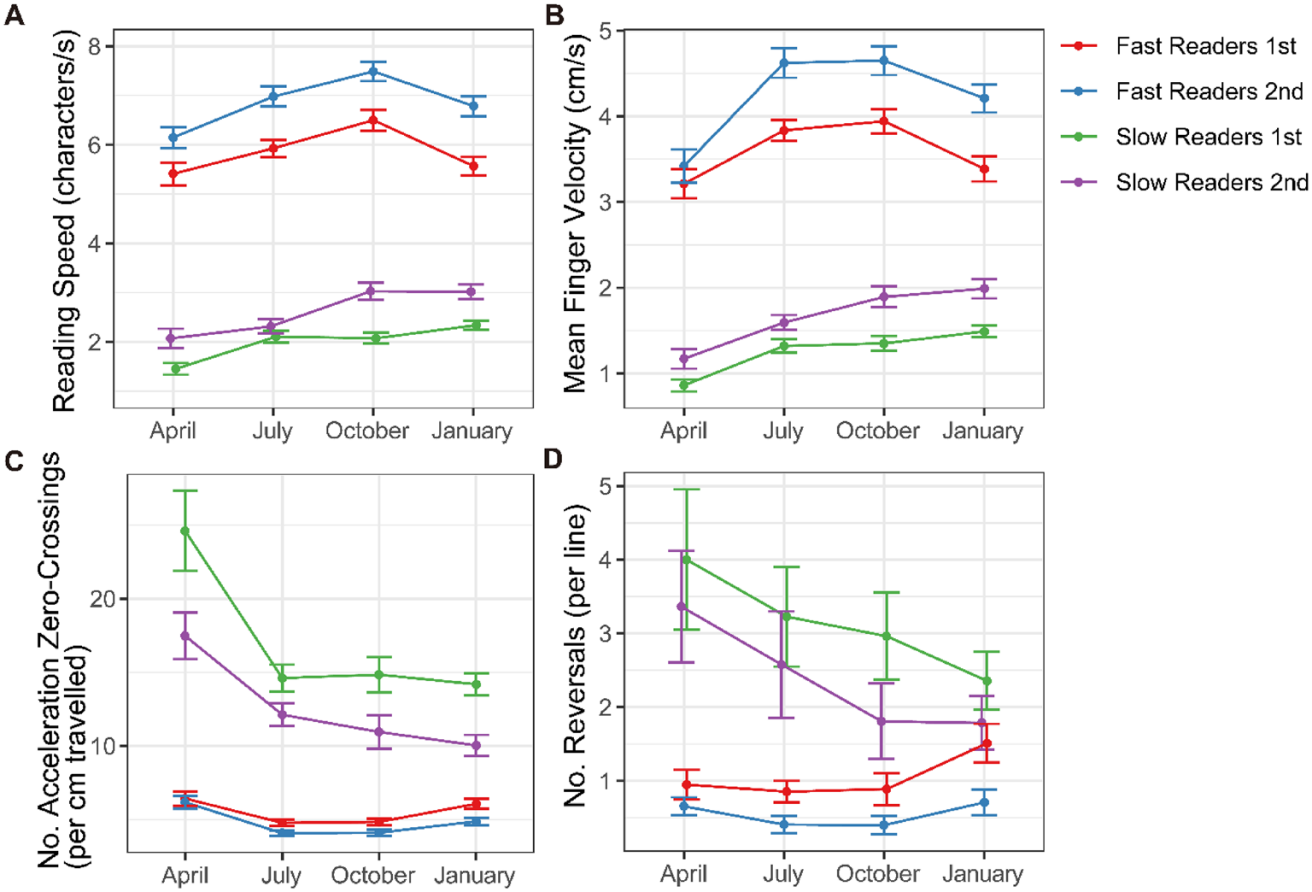
(**A**) Mean reading speed in characters per second, (**B**) mean finger velocity in cm/s, (**C**) number of acceleration zero-crossings per cm travelled, and (**D**) number of reversals per line as a function of month (April, July, October 2019, and January 2020), repetition (1st reading and 2nd reading for each month’s recording session), and group (fast readers with more than three years of braille experience and slow readers with 2 years or less experience). Each variable is computed for each line of the braille passage. The braille passage consists of 11 to 14 lines, and the lines comprising of more than 15 braille characters contributed to the analysis (Table S1). Error bars represent ± 1 standard error of the mean. Figure prepared in R software (version 4.0.2, R Core Team, 2020, Vienna, Austria)^43^.

The results are summarized in Table 2 and Fig. 2. Mean reading speed averaged 5.13 characters per second (cps). The fastest reader (P3) averaged 7.91 cps; the slowest (P4) 2.17 cps (Table 2). Reading error was not frequent, ranging from 0 to 13 words in all trials pooled (out of 382 words for those who had read all the lines). The three participants with less than three years of braille reading experience (P4, P7, and P8) read the braille texts considerably slower and appeared to make errors more frequently compared to the other five participants with three or more years of experience (P1, P2, P3, P5, and P6) (Table 2). Accordingly, we partitioned the participants at a population mean of the reading speed into the two groups: slow readers (P4, P7, and P8) with one or two years of braille reading experience, and fast readers (P1, P2, P3, P5, and P6) with three or more years of experience. A linear mixed effects analysis confirmed that the difference in reading speed between the two groups was statistically significant, *F*_(1,6)_ = 56.46, *p* = 0.0003 (Fig. 2A). In addition, reading speed changed over the course of months, *F*_(1,560)_ = 6.61, *p* = 0.0104). The participants read the braille passage significantly faster in second reading compared to first reading (*F*_(1,560)_ = 104.32 *p* < 0.0001). The degree to which reading speed changed over the course of months or between first and second reading did not differ statistically across slow and fast readers, where neither month nor repetition did not interact with group (*Fs*_(1,560)_ < 3.09, *ps* > 0.07).

**Table 2:** Characteristics of individual participants and their braille reading performance.

Participant	Sex	Age	Years of learning braille	Reading speed in cps (*SD*)	Group	Reading error (total no. words)	Hand movement pattern	Velocity in cm/s (*SD*) of dominant hand	Velocity in cm/s (*SD*) of non-dominant hand
P1	M	11	5	6.29 (1.51)	Fast	1 (382)	*Scissors*	3.61 (1.04)	2.73 (0.99)
P2	M	11	5	5.55 (1.29)	Fast	3 (382)	*Scissors*	3.39 (1.17)	2.66 (1.08)
P3	M	10	4	7.91 (1.43)	Fast	3 (382)	*Scissors*	5.11 (1.44)	3.68 (0.97)
P4	M	11	1	2.17 (0.57)	Slow	13 (278)	*One hand*	1.42 (0.38)	–
P5	M	9	3	5.46 (1.12)	Fast	0 (382)	*Scissors*	3.39 (0.94)	2.93 (0.75)
P6	F	12	6	6.39 (1.31)	Fast	4 (382)	*One hand*	4.34 (0.94)	–
P7	M	7	1	2.41 (0.79)	Slow	5 (382)	*Split*	1.51 (0.58)	1.49 (0.54)
P8	F	8	2	2.65 (1.05)	Slow	8 (303)	*Parallel/split*	1.67 (0.70)	1.66 (0.62)

Age refers to each participant’s age at the beginning of study. Hand movement pattern of P8 was observed to have changed from “parallel” to “split” from the third recording session (October, 2019). Total number of words read by P4 and P8 was small compared to other participants for the following reasons: P4 was unable to read braille sentences at the first recording. P8 read only few lines of the text in the first and second session, which she read only once without repetition. The velocity of the reading finger of the non-dominant hand is shown for reference.

### Lateral scanning movement

Consistent with previous studies^21^, all participants used their index fingers during braille reading. All fast readers used the two-handed “scissors” pattern except one-handed reader P6 (Table 2) (see “Methods” section for categories of hand movement pattern). Among slow readers, P4 used one hand to read, and P7 used the “split” pattern. The hand movement pattern of P8 was observed to have changed from “parallel” to “split” from the third testing session (October, 2019). Informally, we observed, among the two-handed readers, that the difference in scanning speed between the two hands tended to be smaller for slow readers compared to fast readers (Table 2). Presumably, this was because slow readers moved the two hands together until near the end of each line.

**Figure 3:**
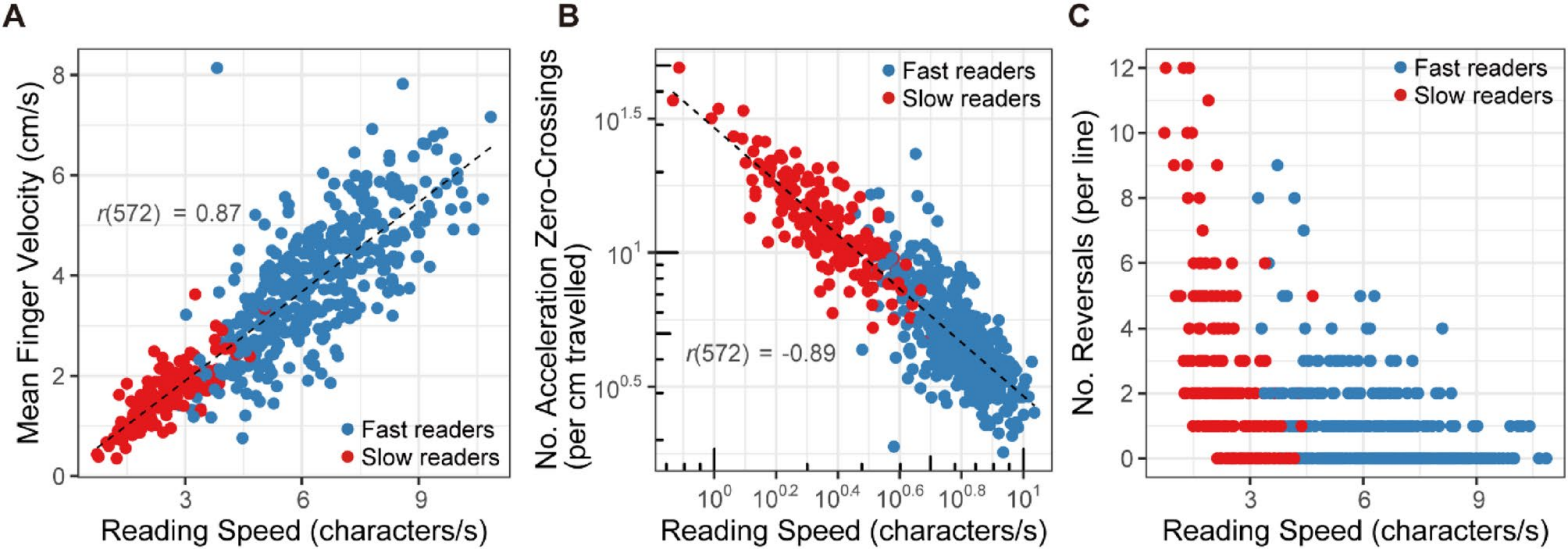
(**A**) The relation between mean finger velocity and reading speed (*r*^*2*^ = 0.77, *p* < 0.0001), (**B**) between number of acceleration zero-crossings and reading speed (*r*^*2*^ = 0.80, *p* < 0.0001), and (**C**) between number of reversals per line (whose values are always integer) and reading speed, colored according to the group (fast readers and slow readers). Recordings over multiple recording sessions and repetitions are pooled. Each variable is computed for each line of the braille passage. Figure prepared in R software (version 4.0.2, R Core Team, 2020, Vienna, Austria)^43^.

Mean velocity of the dominant reading finger in the left–right dimension across all trials averaged 3.17 cm/s (*SE* = 0.064), which was strongly related to the reading speed (*r*_572_ = 0.87, *p* < 0.0001, *CI* = [0.85, 0.89]) (Fig. 3A). Replicating the results of reading speed, participants scanned the braille texts faster from first to second reading (*F*_(1, 560)_ = 71.11, *p* < 0.0001) (Fig. 2B). Fast readers scanned the braille texts faster compared to slow readers (*F*_(1,6)_ = 26.54, *p* = 0.0021) with no interactions with month or trial. The frequency of acceleration zero-crossings revealed that finger movements reading braille did not proceed at constant speed along each line of text. On average, the velocity profiles of fast readers contained as many as 5.23 acceleration zero-crossings per each cm travelled, and those of slow readers 13.94, and the difference between the two groups was significant (*F*_(1,6)_ = 76.62, *p* = 0.0001) (Fig. 2C). Visual inspection of Fig. 2C suggests that slow readers changed over months and over repetition in such a way to decrease the number of acceleration zero-crossings, which was confirmed by significant interactions between group and month (*F*_(1,6)_ = 10.63, *p* = 0.0012), and group and repetition (*F*_(1,6)_ = 30.47, *p* < 0.0001), respectively. The number of acceleration zero-crossings exhibited negative logarithmic correlations to the oral reading speed (*r*_572_ =  − 0.89, *p* < 0.0001, *CI* = [− 0.91, − 0.87]) (Fig. 3B). Since the mean finger velocity scanning each line was strongly related to the oral reading speed (Fig. 3A), this result replicated the finding that the frequency of acceleration zero-crossings decrease exponentially as reading speed and finger scanning velocity increase^22,23^. The other aspect of intermittency—reversal of direction of scanning—averaged 1.34 times per line. The reversal of scanning direction occurred more frequently for slow readers compared to fast readers (*F*_(1,6)_ = 96.10, *p* = 0.0001), and during first reading compared to second reading (*F*_(1,560)_ = 16.12, *p* = 0.0001) (Figs. 2D, 3C). As can be seen from Fig. 2D, the number of reversal decreased across months only for slow readers, which was statistically confirmed by a significant interaction between group and month (*F*_(1,560)_ = 5.70, *p* = 0.0173).

### Variation in angular orientation

**Figure 4:**
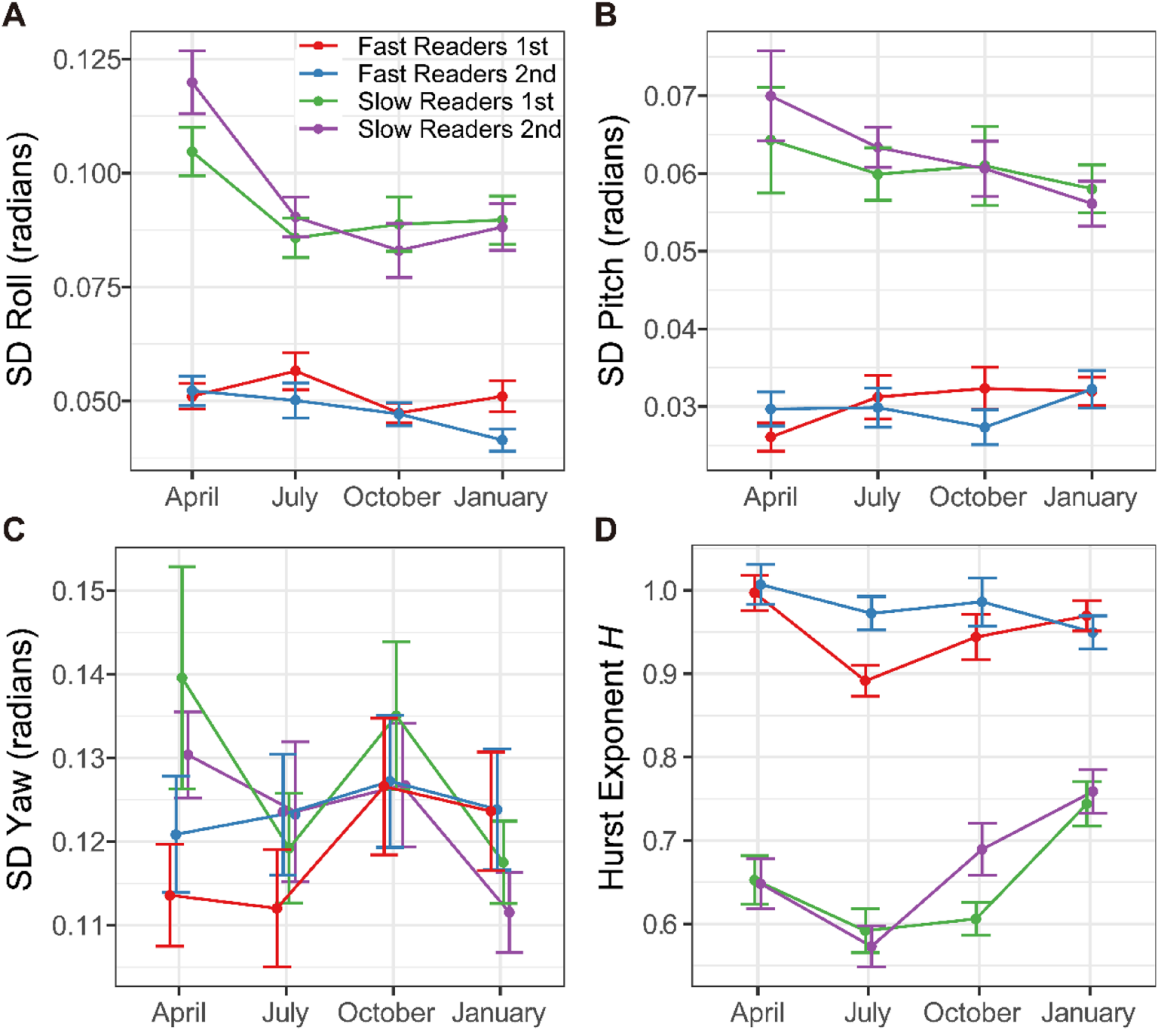
(**A**) Mean angular standard deviations of roll of the reading finger, (**B**) mean angular standard deviations of pitch of the reading finger, (**C**) mean angular standard deviations of yaw of the reading finger, and (**D**) mean Hurst exponent *H* of scanning movement time series as a function of month (April, July, October 2019, and January 2020), repetition (1st reading and 2nd reading for each month’s recording session), and group (fast readers and slow readers). Each variable is computed for each line of the braille passage. Error bars represent ± 1 standard error of the mean. Figure prepared in R software (version 4.0.2, R Core Team, 2020, Vienna, Austria)^43^.

**Figure 5:**
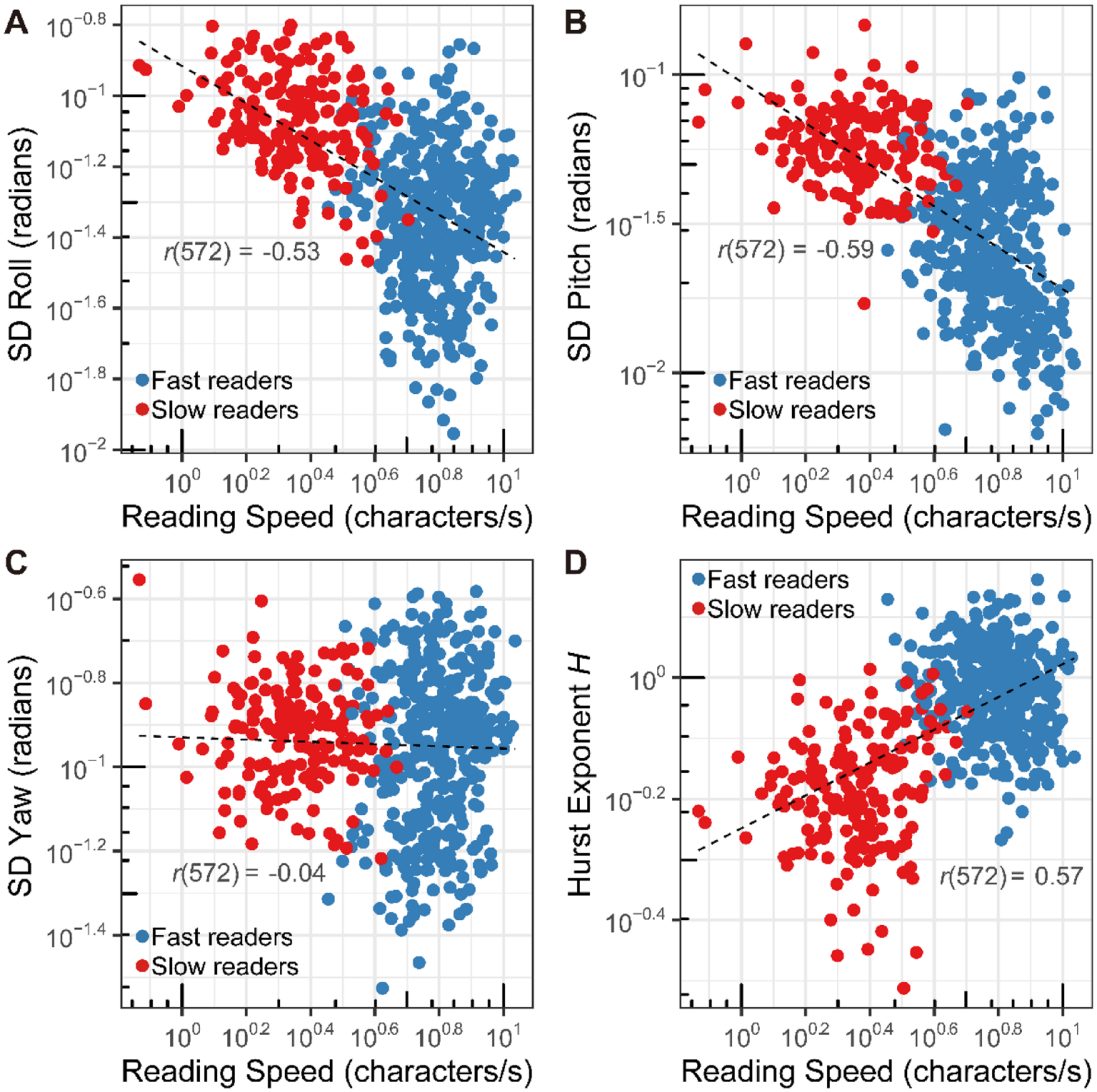
(**A**) The relation between mean angular standard deviations of roll angle of the reading finger and reading speed (*r*^*2*^ = 0.30, *p* < 0.0001), (**B**) between mean angular standard deviations of pitch of the reading finger and reading speed (*r*^*2*^ = 0.38, *p* < 0.0001), (**C**) between mean angular standard deviations of yaw of the reading finger and reading speed (*r*^*2*^ = 0.00 *p* = 0.39), and (**D**) between Hurst exponent *H* of scanning movement time series and reading speed for each line (*r*^*2*^ = 0.32, *p* < 0.0001), colored according to the group (fast readers and slow readers). Recordings over multiple recording sessions and repetitions are pooled. Each variable is computed for each line of the braille passage. Figure prepared in R software (version 4.0.2, R Core Team, 2020, Vienna, Austria)^43^.

The angular standard deviation of the finger orientation in the three axes (roll, pitch, and yaw) during the scanning of each line reflects the variability of the orientation of finger touching the braille paper. In the present experimental setup, roll corresponds to rotation with respect to longitudinal axis of the finger, pitch corresponds to the rotation with respect to transverse axis of the finger, and yaw corresponds to the rotation with respect to vertical axis. The former two angles affect the contact region on the fingerpad, while yaw determines the direction of finger on the paper plane (Fig. 1C). Visual inspection of Fig. 4A and B suggests that the orientation of slow reader’s (P4, P7, and P8) scanning finger in the roll and the pitch axes tended to be more variable compared to those of the fast readers. A linear mixed effects analysis confirmed that the orientation of slow reader’s scanning finger was more variable compared to the fast readers in the roll axis (*F*_(1,6)_ = 13.34, *p* = 0.0107) as well as in pitch (*F*_(1,6)_ = 11.09, *p* = 0.0158) (Fig. 4A,B). The variability of orientation in these two axes did not change across repetitions within a session, or across testing sessions. When log-transformed, reading speed was negatively related to the variability of finger orientation in roll (*r*_572_ =  − 0.53, *p* < 0.0001, *CI* = [− 0.58, − 0.46]) as well as in pitch (*r*_572_ =  − 0.59, *p* < 0.0001, *CI* = [− 0.64, − 0.54]) (Fig. 5A,B), indicating that the invariance of the finger orientation in roll and pitch led to faster oral reading performance. Unlike these two axes, however, variability of finger orientation in yaw was not influenced by groups, repetition, or month (*Fs* < 2.60, *ps* > 0.1), and the difference in the variability of orientation around this axis was not related to braille reading performance (*r*_572_ =  − 0.04, *p* = 0.39, *CI* = [− 0.12, 0.05]) (Figs. 4C, 5C).

### Temporal structure of lateral scanning movement

Sampling of the scanning of 574 lines read by the eight participants at 120 Hz produced 574 time series with a mean length of 613 data points (*SE* = 18.97). Displacement time series were produced by taking the Euclidean distance in left–right dimension between each pair of consecutive points. DFA for each original 574 displacement time series returned Hurst exponents *H* estimates consistent with long-range temporal correlations for original series (*M* = 0.88, *SE* = 0.008), and exceeded *H* for shuffled displacement series (*M* = 0.46, *SE* = 0.004), paired-samples *t*(573) = 44.99, *p* < 0.0001. Visual inspection of Fig. 4D suggests that the scanning movements of fast readers (P1, P2, P3, P5, and P6) exhibited stronger long-range temporal correlations compared to those of slow readers (P4, P7, and P8), which was confirmed by a linear effects model analysis (*F*_(1,6)_ = 40.79, *p* = 0.0007). Group significantly interacted with month, indicating that the pattern of change over months was different between fast and slow readers (*F*_(1,560)_ = 11.70, *p* = 0.0007). As can be seen in Fig. 4D, slow readers tended to develop over months in such a way to have stronger temporal correlations in scanning movement, whereas this was not the case with fast readers. It was also found that between first and second reading, the temporal correlations in scanning movement got slightly stronger (*F*_(1,560)_ = 6.15 *p* = 0.0135). Overall, the temporal correlations in scanning movements were logarithmically related to the reading speed (*r*_572_ = 0.57, *p* < 0.0001, *CI* = [0.51, 0.62]), indicating that the stronger long-range temporal correlations in finger scanning movements led to the faster oral reading performance (Fig. 5D).

### Growth curve modelling for braille reading performance

Using a growth curve modelling technique, the reading speeds were modelled as a weighted sum of main effects and interactions^28,42^. We begin building a model of trial-by-trial braille reading performance with a series of multi-level models of reading speeds. All models appear in terms of the highest available interactions tested, and all lower-order interactions were included implicitly. Starting from the model with month and repetition as fixed effects and individual participant as a random effect for the intercept as well as its slope with respect to month intercept effect, new effects as well as their full-factorial interactions with all previous terms in the model were added successively. Table 3 outlines the details of this sequence. First, we added to the model the number of acceleration zero-crossings, which reflects the overall number of inflection points in the velocity trace^22,23^. This led to a significant improvement in the prediction of reading speed in Model 2, *χ*^2^(19) = 183.83, *p* < 0.0001. In Model 3, we included a term for trial-by-trial changes in *H*, which significantly improved the prediction of reading speed, *χ*^2^(27) = 24.98, *p* = 0.0016, thereby confirming that the temporal structure of lateral scanning movements significantly impacted braille reading performance. In Model 4, we added the angular standard deviation of finger orientation in pitch, which yielded further significant improvement, *χ*^2^(43) = 49.66, *p* < 0.0001. Finally, in Model 5 we included the angular standard deviation of finger orientation in roll, again yielding more accurate prediction, *χ*^2^(75) = 55.62, *p* = 0.0060. Overall, the results suggest that each of these aspects of structure of variability in finger movement may characterize the skill of  braille reading.

**Table 3:** Building of growth curve models in terms of highest-order interaction and chi-square (*χ*^2^) tests for improvements in model fit.

Model	Terms	*χ*^*2*^	*df*	*p*
1	Repetition × Month		15	
2	Model 1 × No. Acceleration Zero-Crossings	183.83	19	< 0.0001
3	Model 2 × Hurst Exponent	24.98	27	0.0016
4	Model 3 × SD Pitch	49.66	43	< 0.0001
5	Model 4 × SD Roll	55.62	75	0.0060

In addition, participant was incorporated as a random effect for the intercept as well as its slope with respect to month into all the models.

## Discussion

In this study, we asked whether kinematic characteristics of scanning movements of the reading finger might be related to the function of braille reading. We compared inexperienced slow readers and more experienced fast readers at four points during a single year. Our results indicated that the strength of long-range temporal correlations in lateral scanning movements of the reading finger correlated with braille reading performance, and that invariance of the orientation of the reading finger predicted braille reading performance.

We replicated previous research^22,23^, in which lateral scanning movement of the reading finger was not at constant speed but involved not only acceleration (both positive and negative) but also reversals of direction. We also replicated the finding that the mean instantaneous velocity of the reading finger was negatively correlated with the number of acceleration zero-crossings^22,23^. Our analysis of the temporal structure of scanning movements revealed several novel findings. First, the velocity variation of scanning movements was not random, but exhibited long-range power-law temporal correlations. Second, confirming our first hypothesis, the long-range temporal correlation properties in lateral scanning movements of the reading finger predicted braille reading performance. That is, stronger temporal correlations were associated with faster reading speed. Third, for slower, less-experienced readers, the lateral scanning movement of the reading finger was found to develop over months such that the strength of temporal correlations across time scales tended to increase.

Taken together, our results open new perspectives in understanding the role of movement variability in lateral scanning during braille reading. What would be the possible functional role of such movement fluctuations? Previous researchers have claimed that braille reading would be optimized when the scanning finger moved at constant speed^44^. Implicit in this view is the assumption that variability degrades perceptual sensitivity. An alternative view suggests a qualitatively different hypothesis; that movement variability may contribute to separating off the invariant information about the adjacent environment over transformations of stimulus patterns^9,10,27–31^. In this view, variations in movement of the scanning finger might be controlled so as to efficiently isolate informative structures that specify the braille text.

A growing body of evidence now indicates that fluctuations in exploratory movement exhibit long-range power-law temporal correlations, in which the fluctuations grow faster than would be expected with random uncorrelated noise^27–29^. One interpretation of such evidence is that long-range temporal correlations produce rapidly growing fluctuations that contribute to efficient search for informative structures that specify the external source^25,45^; in the present case, the pattern of raised dots on the page. Such temporal structure in movement might be achieved through perturbation by an active perceiver of the interconnected structural hierarchies of our bodies—from skins and other connective tissues to various micro-elastic structures such as a network of collagen fibers^30,31,46,47^. Our results cast doubt on the claim that braille readers associate central and peripheral signals to internally compensate for variations in finger speed^48^. The presence of nonlinearity arising from interaction across fluctuations at different time scales, as documented in the present study, would greatly complicate such a process. Our results are more compatible with contemporary concepts of active touch, in which perception depends on exploratory movements that differentiate the invariant patterns that specify the source of mechanical disturbances from all the other patterns that do *not* specify the source^10,30,31^.

We also confirmed our second hypothesis. Underlying lateral scanning intermittencies, angular adjustments of the reading finger maintained a certain invariant finger orientation relative to the braille dots. The degree of this invariance was significantly related to braille reading performance. Of the three angles that we measured, roll and pitch varied the contact region on the fingerpad, while yaw reflected the direction of the finger on the paper plane. Our results revealed that the degree of invariance of roll and pitch was related to braille reading performance. This means that, during continuously fluctuating scanning movements, the posture of the fingerpad was stabilized in such a way as to contact braille dots with more or less invariant regions of the fingerpad. The presence of fine adjustments of receptor surfaces is not limited to active touch, but has been found in a variety of contexts^37–41^. Similarly, our results suggest that in braille reading the control of orientation of the small, high resolution area may play a functional role in the efficient discrimination of tactile information.

How are the kinematic characteristics of scanning movements of the reading finger related to the skill of braille reading? Contrary to previous interpretations, we found that the long-range power-law temporal correlations in the velocity fluctuations of lateral scanning movement were functionally related to braille reading performance and its development. We also found that variation in angular orientation of the reading finger was related to braille reading performance. Our results provide the first experimental evidence that the structure of variability in scanning movements of the reading finger are related to the functional performance in braille reading.

## Methods

### Participants

The participants were eight congenitally or early blind pupils, including 2 females and 6 males. Each participant was enrolled in one of two schools for visually impaired children (Kobe, Japan). At the time of the first recording session, their ages ranged from 7 to 12 years. At the time of the first recording session, the participants had from 1 to 6 years of braille reading experience. Their legal guardians provided informed consent in accordance with the Declaration of Helsinki. This research was conducted in compliance with protocols approved by the Institutional Review Board of Graduate School of Human Development and Environment, Kobe University.

### Materials and apparatus

As reading materials for our study, we selected four passages of prose from introductory reading textbooks intended for sighted students. The reading materials were converted to braille notation using a software *T Editor 3* (t-editor.sakura.ne.jp). The converted texts were subsequently proofread by teachers. We sought to ensure that all participants could understand the reading passages and, therefore, selected texts from textbooks intended for students in the first grade. Teachers reviewed the selected passages to confirm that they were easy enough to read, and that students had not already read them as part of their classwork. Using a Perkins Brailler (Perkins Solutions, Watertown, MA), each passage was printed with single line spacing on one B5 size standard sheet of braille paper. There were between 11 and 14 lines in each passage. The number of braille characters ranged from 4 to 27 per line, for a total of from 242 to 286 braille characters per passage (Supplementary Table S1). Line breaks occurred at natural breaks in the text. A different passage was used for each of the tri-monthly testing periods.

Based on the previous reports that braille readers primarily use the two index fingers to read braille^21^, we recorded the kinematics of the index finger on each hand during braille reading using an magnetic tracking system (Polhemus G4). In this system, a stationary emitter generated a magnetic field. Sensors created spatial distortions in the field, which were detected by the system, yielding quantitative data about the position of sensors within the field in six degrees of freedom (3 axes of translation, and 3 axes of rotation). The linear accuracy of the system was 0.2 cm, while it’s angular accuracy was 0.5°. One sensor (Polhemus Micro Sensor 1.8) was firmly attached with adhesive tape to the fingernail of each index finger (Fig. 1B). Each sensor weighed less than one gram. Movement data were recorded at 120 Hz. The magnetic field emitter was positioned so that the linear distance to each sensor never exceeded 0.7 m. The participants reported that the sensors were not a hindrance. We made digital video recordings of each trial, using an overhead camera whose view captured the movement of the fingers on the braille sheet.

### Procedure

Data collection took place in April, July, and October of 2019, and January of 2020 (Supplementary Table S1). Each individual participated in all four sessions. Participants were tested individually. Data were collected in a quiet classroom in each pupil’s school. Participants used the same desks and chairs they regularly used in their classroom activities. The participant was presented with a sheet of braille paper attached to the desk using adhesive tape, and was instructed to read the entire page aloud. At each testing session, participants read the same passage twice. There was a brief pause between 1st and 2nd reading, in which the children waited until they were told to read again. Each session lasted about 15 min including the time required to attach the sensors to the fingers.

### Data analysis

Movement kinematics were analyzed separately for each line of text. To ensure an adequate number of data points for analysis, we included data only from lines that comprised more than 15 braille characters (Supplementary Table S1). We analyzed reading performance by computing reading speed for each line of text. We identified the reading errors based on the video recordings. We analyzed movements corresponding to individual lines, that is, we excluded movement related to the repositioning of the hands from the end of one line to the beginning of the next. We analyzed for the left–right axis, corresponding to the movement of the hand laterally across the text. Separately, we analyzed the orientation of the index finger in three axes of rotation (Fig. 1C). As a measure of the variability of the orientation of the index fingers, we computed angular standard deviations of three angular coordinates (roll, pitch, and yaw). Roll and pitch affected the contact region on the fingerpad, while yaw determined the direction of finger on the paper plane. The raw position and angle data were low-pass filtered (set at 12 Hz) with a dual-pass fourth order Butterworth filter.

Some participants used both hands in braille reading, that is, they simultaneously used the index finger of both hands. We focused on the dominant reading finger, which we operationally defined as the finger that covered the greater number of characters. If the dominant reading finger changed within the same participant across different lines or different recording sessions, the finger that was dominant in greater number of lines across all recording sessions was defined as the dominant reading finger.

We used the audio from video recordings to determine the duration of oral reading of individual lines of text by a video coding software *Datavyu* (datavyu.org). Following Legge et al.^49^, we computed the reading speed for each line of text, in terms of characters per second (cps). Punctuation and spaces were included in the character count, and two consecutive spaces were coutned as a single space. Data were included even in cases in which a participant misread or skipped a word in a line. We recorded the number of errors made by each participant in each session. If a participant did not finish reading a line, the data from the line was excluded from the analysis.

The hand movements observed were classified into the five categories used by Wright et al^50^: (1) *One-handed*: only one hand was used for reading, (2) *one marks*: one hand was used a marker while the other read, (3) *parallel*: both hands read together, (4) *split*: both hands read together until near the end of the line, at which they split with one hand returning to locate the next line, (5) *scissors*: the left hands read to the middle of the line where right hand joins it, and the right hand finished reading the line.

### Dependent variables

We evaluated braille reading performance in terms of reading speed, measured in characters per second (cps). We characterized the movement of the reading finger using several parameters (Table 1): (1) mean lateral (i.e., left to right) finger scanning velocity (cm/sec), (2) the number of acceleration zero-crossings per cm travelled, (3) the number of distinct reversals executed in each line, where a reversal was defined as the transition from positive to negative velocity in the left–right dimension over more than 0.5 cm which is followed by a negative-to-positive transition, (4) the Hurst exponent *H* of lateral finger position time series obtained from detrended fluctuation analysis, and (5) angular standard deviations (in radians) of the index finger in the three three axes (radians).

### Detrended fluctuation analysis (DFA)

In DFA method^33^, first, the original time series *x*(*t*) (with *N* samples) is shifted by the mean $\stackrel{-}{x\left(t\right)}$ and integrated to produce the new time series *y*(*t*):

1 $$y(t)=\sum_{i=1}^{N}[x\left(i\right)-\stackrel{-}{x\left(t\right)}]$$

Next, the integrated time series is divided into windows of equal length, *n*. For each window of length *n*, a least squares line is fit to the data. The *y* coordinate of the straight-line segments is denoted by *y*_*n*_(*t*). For a given window size *n*, the characteristic size of the fluctuations, denoted by *F*(*n*), is then calculated as the root-mean-square deviation between *y*(*t*) and its trend *y*_*n*_(*t*) in each box:

2 $$F\left(n\right)=\sqrt{\frac{1}{N}\sum_{i=1}^{N}{[y\left(t\right)-{y}_{n}\left(t\right)]}^{2}}$$

This computation is repeated over all window sizes. The slope of the line relating log *F*(*n*) to log *n* determines the scaling exponent, *H*:

3 $$F\left(n\right)\sim {n}^{H}$$

We used a first-order differentiation of lateral scanning trajectories as a data *x*(*t*), which can be classified as fractional Gaussian noise^36^. If *H* = 0.5, there is no correlation and the time series is uncorrelated random noise. If *H* > 0.5, the time series is correlated, and the closer the value of *H* to 1, the stronger the long-range temporal correlations in the time series^36^. If *H* < 0.5, the time series is anti-correlated with the large fluctuations at short time scales and the lack of large fluctuations at large time scales. To validate that the obtained *H* is associated with long-range temporal correlations in the fluctuations of the measured time series, we performed DFA on randomly shuffled versions of each of the original 574 displacement time series, as well as on the original time series. A signal that truly exhibits the property of long-range temporal correlations will bear a scaling exponent *H* > 0.5 in its original sequence but not in a shuffled sequence.

### Statistical analysis

For all statistical tests, we used criterion α = 0.05 (two-sided). Before conducting statistical analysis, we partitioned the participants into fast and slow readers using a mean split of the reading speed. The grouping based on reading speed turned out to correspond exactly to the grouping based on the amount of braille reading experience, where fast readers corresponded to the children with three or more years of braille experience, and slow readers corresponded to those with one or two years of braille experience, respectively.

We conducted two separate analyses. The aim of the first analysis is to characterize the difference in the scanning movement of the reading finger between inexperienced slow readers and more experienced fast readers, between different times of the year during which learning took place, and between repeated trials within a session. Using the *lme* function in the *nlme* package^51^ of the R statistical software^43^, aforementioned measures of braille reading performance were modelled using a linear mixed effects model^52^. The fixed effects factors were months (April, July, October, January), repetition (first vs. second reading), and group (fast vs. slow readers). Participants was included as a random effect for the intercept as well as its slope with respect to month.

Second, using a growth curve modelling technique, we modelled the change in reading speed as a weighted sum of main effects and interactions^28,42^. Starting from the model with month and repetition as fixed effects and individual participants as a random effect for the intercept as well as its slope with respect to month, we tested the effects of adding the number of acceleration zero-crossings (i.e., the overall number of inflection points in the velocity trace)^22,23^, Hurst exponent *H*, and angular standard deviations of the roll and pitch angles of the orientation of the index finger as predictors by treating the change in a deviance measure (i.e., − 2 log likelihood) as a chi-square statistic. To model heteroscedasticity, we used a variance function (*varIdent* of *nlme* package) that allowed different variances per stratum for individual participants^51^.

## Supplementary Information

Supplementary Informations.

## Data Availability

All data are available on reasonable request from the corresponding author.
