# Supplementary material for: Structure of variability in scanning movement predicts braille reading performance in children
Source: Sci Rep. 2021 Mar 30;11:7182. doi: 10.1038/s41598-021-86674-5 (PMC8009883; doi:10.1038/s41598-021-86674-5)
Supplement: Supplementary file 1 — Supplementary Informations. [file 41598_2021_86674_MOESM1_ESM.docx]

*****Supplementary Information*****

**Structure of variability in scanning movement predicts braille reading performance in children**

Tetsushi Nonaka^1*^, Kiyohide Ito^2^, and Thomas A. Stoffregen^3^

**Author affiliations**

^1^ Graduate School of Human Development and Environment, Kobe University, Kobe, Japan.

^2^ School of System Information Science, Future University Hakodate, Hakodate, Japan.

^3^ School of Kinesiology, University of Minnesota, Minneapolis, MN, USA.

***Corresponding author and lead contact: Tetsushi Nonaka**

Complete address: Graduate School of Human Development and Environment, Kobe University, Tsurukabuto 3-11, Nada-ku, Kobe, 6578501, Japan

Phone: +81 78 803 7801

Email: tetsushi@people.kobe-u.ac.jp

**Supplementary Table S1**. Schedule of longitudinal recording sessions and reading materials used in each recording session

| Recording Session | Reading materials | | |
| --- | --- | --- | --- |
|  | No. lines (characters) | No. lines (characters) contributed to analysis | Mean reading duration |
| 1 (April, 2019) | 14 (286) | 12 (272) | 81 s (min: 35 s, max: 233 s) |
| 2 (July, 2019) | 11 (242) | 10 (236) | 73 s (min: 28 s, max: 215 s) |
| 3 (October, 2019) | 11 (244) | 9 (218) | 65 s (min: 28 s, max: 156 s) |
| 4 (January, 2020) | 12 (258) | 10 (248) | 72 s (min 33 s, max: 140 s) |
